# Systematic Understanding of the Mechanism of Baicalin against Ischemic Stroke through a Network Pharmacology Approach

**DOI:** 10.1155/2018/2582843

**Published:** 2018-12-17

**Authors:** Tian Xu, Chongyang Ma, Shuning Fan, Nang Deng, Yajun Lian, Ling Tan, Weizhe Du, Shuang Zhang, Shuling Liu, Beida Ren, Zhenhan Li, Qinguo Wang, Xueqian Wang, Fafeng Cheng

**Affiliations:** School of Traditional Chinese Medicine, Beijing University of Chinese Medicine, Beijing 100029, China

## Abstract

Ischemic stroke is accompanied by high mortality and morbidity rates. At present, there is no effective clinical treatment. Alternatively, traditional Chinese medicine has been widely used in China and Japan for the treatment of ischemic stroke. Baicalin is a flavonoid extracted from Scutellaria baicalensis that has been shown to be effective against ischemic stroke; however, its mechanism has not been fully elucidated. Based on network pharmacology, we explored the potential mechanism of baicalin on a system level. After obtaining baicalin structural information from the PubChem database, an approach combined with literature mining and PharmMapper prediction was used to uncover baicalin targets. Ischemic stroke-related targets were gathered with the help of DrugBank, Online Mendelian Inheritance in Man (OMIM), Genetic Association Database (GAD), and Therapeutic Target Database (TTD). Protein-protein interaction (PPI) networks were constructed through the Cytoscape plugin BisoGenet and analyzed by topological methods. Gene ontology (GO) and Kyoto Encyclopedia of Genes and Genomes (KEGG) pathway enrichment were carried out via the Database for Annotation, Visualization, and Integrated Discovery (DAVID) server. We obtained a total of 386 potential targets and 5 signaling pathways, including mitogen-activated protein kinase (MAPK), phosphoinositide 3-kinase (PI3K)/protein kinase B (AKT), hypoxia-inducible factor-1 (HIF-1), nuclear factor kappa B (NF-*κ*B), and forkhead box (FOXO) signaling pathways. GO analysis showed that these targets were associated with antiapoptosis, antioxidative stress, anti-inflammation, and other physiopathological processes that are involved in anti-ischemic stroke effects. In summary, the mechanism of baicalin against ischemic stroke involved multiple targets and signaling pathways. Our study provides a network pharmacology framework for future research on traditional Chinese medicine.

## 1. Introduction

A stroke is a common medical condition among adults worldwide, characterized by high mortality, morbidity, and disability rates [1]. Ischemic stroke accounts for 88% of stroke cases [2] and is defined as a condition in which cerebral vessels are blocked and blood perfusion to the brain is decreased, leading to poor oxygen and glucose supplies [3]. The two major therapeutic strategies for ischemic stroke are thrombolytic therapy and neuroprotective therapy [4]. So far, recombinant tissue plasminogen activator (rtPA) is the only compound approved by the Food and Drug Administration as an effective strategy against acute ischemic stroke [5]. Due to time window limitations and other difficulties, potential drugs for ischemic stroke treatment are urgently required. It is well known that multiple pathologic processes are involved in ischemic stroke, including energy metabolism and oxidative stress that vary as the ischemia-reperfusion injury progresses [6]. To address this, future interventions should focus on combination drugs and multitarget compound development.

Traditional Chinese medicine has been shown to be effective in promoting recovery after ischemic stroke [7]. Huanglian-Jie-Du-Tang (HLJDT), a classic traditional Chinese herbal formula consists of four herbs such as Coptidis Rhizoma, Scutellariae Radix, Phellodendri Chinensis Cortex, and Gardeniae Fructus at the weight ratio of 3:2:2:3, has been widely used to treat ischemic stroke in clinical settings in China and other Asian countries [8]. During studying the effective substances of HLJDT against ischemic stroke, our team highlighted baicalin, a flavones compound isolated from HLJDT, for its beneficial effects against ischemic stroke. Consistent with the observation from other researchers [9–11], our previous data showed that baicalin exhibited neuroprotective effects in middle cerebral artery occlusion (MCAO) rats within a therapeutic time window of 4 h and had good antioxidative effects in vivo and in vitro [12].

As the most significant compound discovered from HLJDT, baicalin exhibits definite biological activity. However, research on the mechanism of baicalin anti-ischemic stroke effects remains at the level of animal indicators, while the absence of a systemic or holistic angle leads to a knowledge gap of understanding multitarget agents such as baicalin. Using systematic methods, network pharmacology expounds the relationships between drugs, targets, and diseases and presents a network of drug targets visually from a holistic perspective [13]. It helps understanding the polypharmacology of a drug and its effect on biological networks to improve efficacy [14]. In the present study, we used a network pharmacology platform, including drug target prediction, protein-protein interaction (PPI) network construction, topology screening, and gene functional analysis to uncover the mechanism of baicalin on ischemic stroke and pinpoint its medicinal value. It offers a novel researching approach for mapping the antisichemic stroke mechanisms of baicalin and identifying potential protein targets that coordinated to produce synergetic effect.

## 2. Materials and Methods

### 2.1. Obtaining Baicalin Structural Information

Structural information of baicalin (PubChem CID: 64982) was obtained from the NCBI PubChem (https://pubchem.ncbi.nlm.nih.gov/) and ZINC databases (http://zinc.docking.org/) [15, 16]. Absorption, distribution, metabolism, and excretion (ADME) screening criteria for baicalin included bioavailability (OB), drug-likeness (DL), blood-brain barrier (BBB), and others. Values were obtained from the Traditional Chinese Medicine Systems Pharmacology (TCMSP) database [17].

### 2.2. Predicting Baicalin-Associated Targets

To identify the potential targets of baicalin, two drug target predictions were carried out. For the first, known targets were derived from the literature from PubMed Central of the NCBI database (http://www.ncbi.nlm.nih.gov/pubmed/). The search terms included “baicalin” and “cerebral ischemia,” “cerebral infarction,” “brain ischemia,” or “stroke”. The second part was derived from the PharmMapper database (http://lilab.ecust.edu.cn/pharmmapper/), which was designed to identify potential targets for small molecules through a reverse pharmacophore mapping approach [18]. A MOL2 file of baicalin was uploaded into the web server and the Human Protein Targets Only database was selected. The top 300 potential targets were obtained and sorted by fit score value.

### 2.3. Mining Known Ischemic Stroke Associated Targets

Known targets related to ischemic stroke were obtained from the four currently available databases using “cerebral ischemia” and “ischemic stroke” as the keywords. The databases employed were DrugBank (http://www.drugbank.ca/; version: 4.3), Online Mendelian Inheritance in Man (OMIM; http://www.omim.org/; last updated: 10th Apr. 2016), Genetic Association Database (GAD; http://geneticassociationdb.nih.gov/; last updated: 1st Sep. 2014), and Therapeutic Target Database (TTD; https://db.idrblab.org/ttd/; last updated: 10th Sep. 2015).

### 2.4. Constructing PPI Networks

PPI data were obtained via the Cytoscape plugin BisoGenet [19], which integrates existing data from six PPI databases, including the IntAct Molecular Interaction Database (IntAct), the Biological General Repository for Interaction Datasets (BioGRID), the Biomolecular Interaction Network Database (BIND), the Molecular INTeraction Database (MINT), the Human Protein Reference Database (HPRD), and the Database of Interacting Proteins (DIP). Two PPI interactive networks were constructed and visualized by Cytoscape software that included predicted baicalin targets and known cerebral ischemia targets. After merging these two networks as a candidate network according to the intersection of PPI data, topological features were analyzed to screen a core PPI network.

### 2.5. Analyzing Network Topological Features

By calculating the six measures, betweenness centrality (BC), degree centrality (DC), eigenvector centrality (EC), closeness centrality (CC), network centrality (NC), and local average connectivity (LAC) with the plugin CytoNCA, the topological properties of every node in the interaction network were analyzed. The definitions and computation equations of these six parameters represent the topological importance of a node in the network. More important nodes receive higher quantitative values within the network [20].

### 2.6. Recognizing Clusters of the Core PPI Network

Densely connected regions in large PPI networks that may represent molecular complexes are defined as topological modules or clusters [21, 22] that have pure network properties. Aggregations of nodes of similar or related function in the same network are called functional modules. A group of network components that together disrupt cellular function and then result in a particular disease phenotype are disease modules. Because topology, functional, and disease modules have the same meaning in the network, the functional module is equal to the topology module and the disease can be regarded as a disturbance and destruction of the functional model. Clusters of core PPI networks were obtained by analyzing the corresponding networks by MCODE, a Cytoscape plugin [21].

### 2.7. Gene Ontology (GO) and Pathway Enrichment Analysis

GO analysis divided into three categories of putative targets, namely biological processes (BP), cellular component (CC), and molecular function (MF), and Kyoto Encyclopedia of Genes and Genomes (KEGG) signaling pathway analysis were carried out using the Database for Annotation, Visualization and Integrated Discovery (DAVID) [23]. A P value ≤ 0.05 was considered significant and enriched GO terms were identified using the hypergeometric test. A bubble chart was plotted using the OmicShare platform, a free online platform for data analysis (http://www.omicshare.com/tools).

## 3. Results

### 3.1. Baicalin Structural Information

With the help of TCMSP, we found some important information related to ADME, such as human OB, DL, Caco-2 permeability, BBB permeability, and Lipinski's rule of five (MW, AlogP, TPSA, Hdon, and Hacc). ADME-related properties of baicalin were inspected by TCMSP comprehensively and, in particular, the DL of baicalin was calculated as 0.75, indicating that baicalin is similar to known drugs (Supplementary Table 1). The following cut-offs, MW < 500 Da, ALogP < 5, DL > 0.18, OB > 30%, and BBB permeability > 0.3, were used to determine the potential drug properties of baicalin. The ADME properties of baicalin fit the above cutoffs except for BBB.

### 3.2. Predicting Baicalin-Associated Targets and Mining Known Ischemic Stroke Associated Targets

Using PharmMapper, we obtained the top 300 potential human protein targets of baicalin (Supplementary Table 2), which are derived from 7302 pharmacophore models. By means of the four available resources, namely, TTD, OMIM, GAD, and DrugBank databases, we obtained 15, 249, 58, and 21 ischemic stroke-related targets, respectively (Supplementary Table 3). Further, 30 overlapping protein targets were recognized from the above two categories of targets.

### 3.3. Constructing PPI Networks and Analyzing Network Topological Features

**Figure 1:**
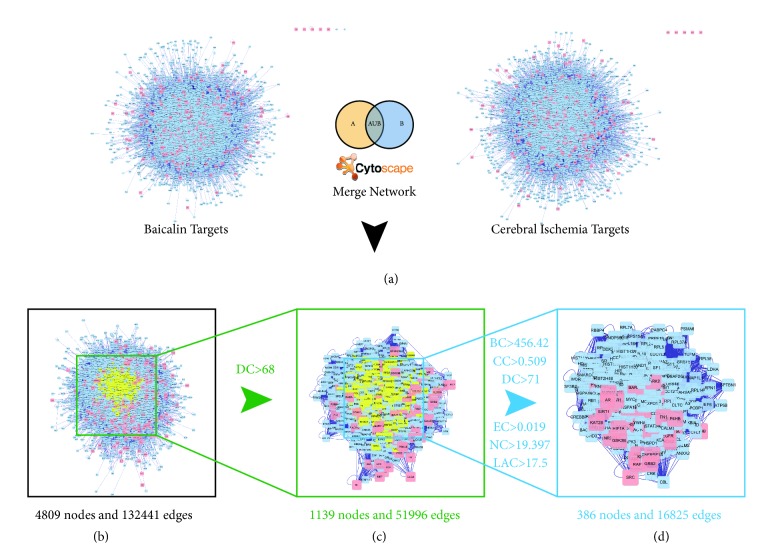
**Identification of a core PPI network for baicalin against ischemic stroke**. (a) Construction of two PPI networks of baicalin targets and ischemic stroke targets. (b) The interactive PPI network of baicalin and ischemic stroke targets comprising 4809 nodes and 132441 edges is shown. (c) PPI network of significant proteins extracted from (b); this network comprises 1139 nodes and 51966 edges. (d) PPI network of significant proteins extracted from (c); this network is made up of 386 nodes and 16825 edges. BC: betweenness centrality; CC: closeness centrality; DC: degree centrality; EC: eigenvector centrality; NC: network centrality; LAC: local average connectivity.

Network pharmacology-based analysis is important in systems biology studies because PPI networks can be used to understand the function of diverse proteins in complex diseases such as ischemic stroke. Thus, we constructed a baicalin target network and an ischemic stroke-related target network using PPI data (Figures 1(a) and 1(b)). To reveal the pharmacological mechanisms of baicalin against ischemic stroke, we used the merge network function provided by Cytoscape to construct a new network (44809 nodes and 132441 edges) consisting of overlapping targets from the two networks (Figure 1(b)). Nodes with degrees only more than twice the median degree of all nodes can be significant targets; therefore, we constructed a network of meaningful targets for baicalin against ischemic stroke with 1139 nodes and 51996 edges (Figure 1(c)). Finally, we selected six topological features to confirm candidate targets, BC, CC, DC, EC, NC, and LAC, with CytoNCA. The candidate targets showed BC values of > 456.42, CC > 0.509, DC > 71, EC > 0.019, NC > 19.397, and LAC > 17.5 (Figure 1(d)).

### 3.4. Enrichment Analysis of Candidate Targets for Baicalin against Ischemic Stroke

**Figure 2:**
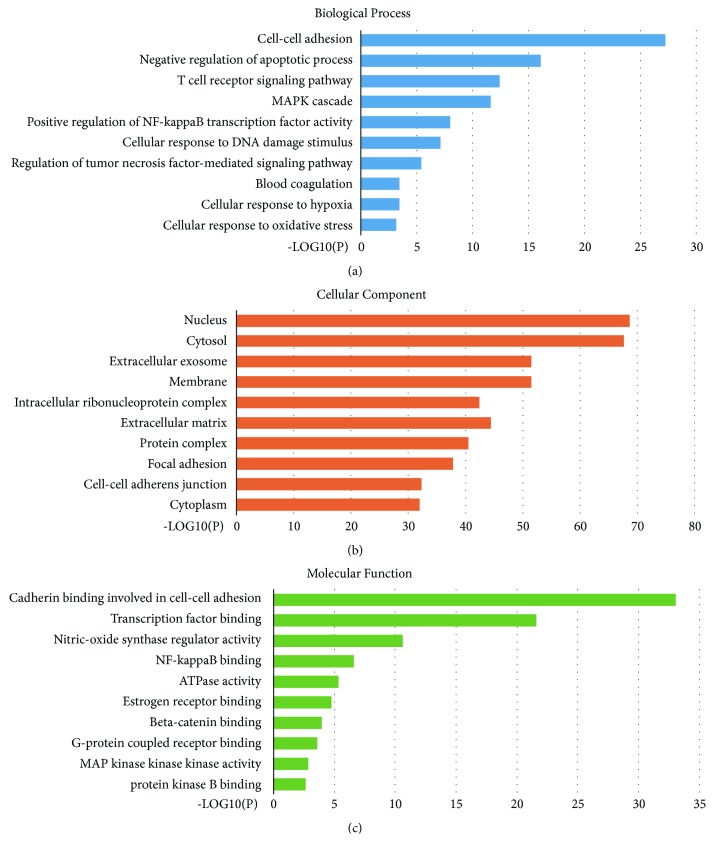
**GO analysis was performed on screened genes**. The top 10 terms for (a) biological processes, (b) cell component, and (c) molecular function with P < 0.05 are shown.

GO analysis of 386 candidate targets for baicalin against ischemic stroke was performed using the DAVID database to understand the relationship between functional units and their underlying significance in the biological system networks. The result was divided into three parts, biological processes (Figure 2(a)), cellular component (Figure 2(b)), and molecular function (Figure 2(c)). We found that biological processes were related to activation of cell-cell adhesion, negative regulation of apoptotic process, T cell receptor signaling pathway, mitogen-activated protein kinase (MAPK) cascade, positive regulation of nuclear factor kappa B (NF-*κ*B) transcription factor activity, cellular response to DNA damage stimulus, regulation of tumor necrosis factor- (TNF-) mediated signaling pathway, blood coagulation, cellular response to hypoxia, and cellular response to oxidative stress. The cellular component was related to the nucleus, cytosol, extracellular exosome, membrane, intracellular ribonucleoprotein complex, extracellular matrix, protein complex, focal adhesion, cell-cell adherens junction, and cytoplasm. Finally, molecular function was related to cadherin binding involved in cell-cell adhesion, transcription factor binding, nitric-oxide synthase regulator activity, NF-*κ*B binding, ATPase activity, estrogen receptor binding, beta-catenin binding, G-protein coupled receptor binding, MAP kinase activity, and protein kinase B (AKT) binding.

### 3.5. Recognizing Clusters of the Core PPI Network

According to ischemic stroke etiology, biological processes can be divided into 8 modules (Figure 4) as follows: (1) cell-cell adhesion (P = 1.9E-5), (2) blood coagulation (P = 3.25E-15), (3) negative regulation of apoptotic process (P = 6.84E-5), MAPK cascade (P = 7.38E-5), and I-*κ*B kinase/NF-*κ*B signaling (P = 0.001), (4) cell-cell adhesion (P = 9.3E-4) and cellular response to interleukin-4 (P = 0.002), (5) neuron apoptotic processes (P = 0.004) and DNA repair (P = 0.004), (6) platelet degranulation (P = 3.86E-5) and regulation of nitric-oxide synthase activity (P = 1.17E-4), (7) cellular response to hydrogen peroxide (P = 0.005), and (8) T cell receptor signaling pathway (P = 6.35E-5) and NF-*κ*B-inducing kinase (NIK)/NF-*κ*B signaling (P = 1.25E-4).

### 3.6. Gene Ontology and Pathway Enrichment Analysis

**Figure 3:**
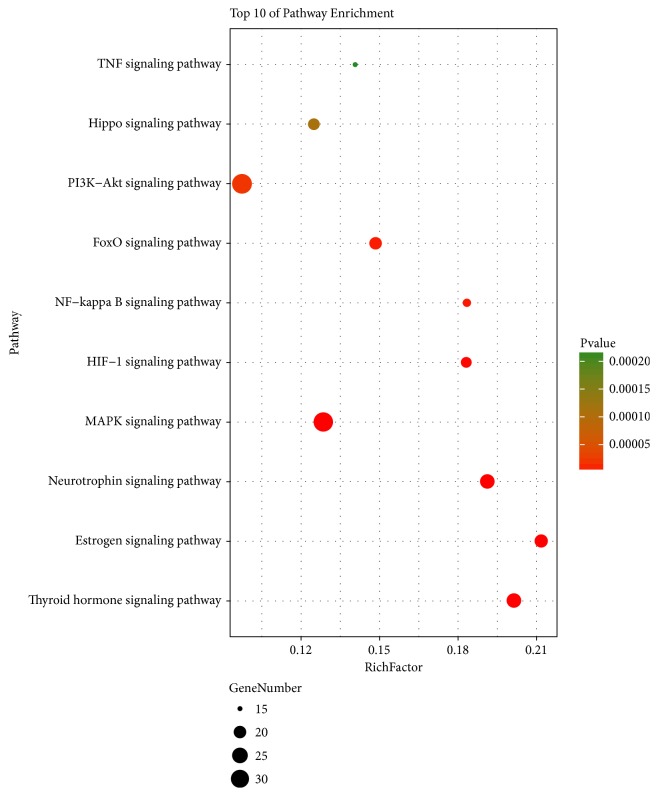
**KEGG signaling pathway enrichment of screened genes**. “Rich factor” represents the ratio of the number of target genes belonging to a pathway and the number of the annotated genes located in the pathway. A higher rich factor represents a higher level of enrichment. The size of the dot indicates the number of target genes in the pathway and the color of the dot reflects the different P values.

**Figure 4:**
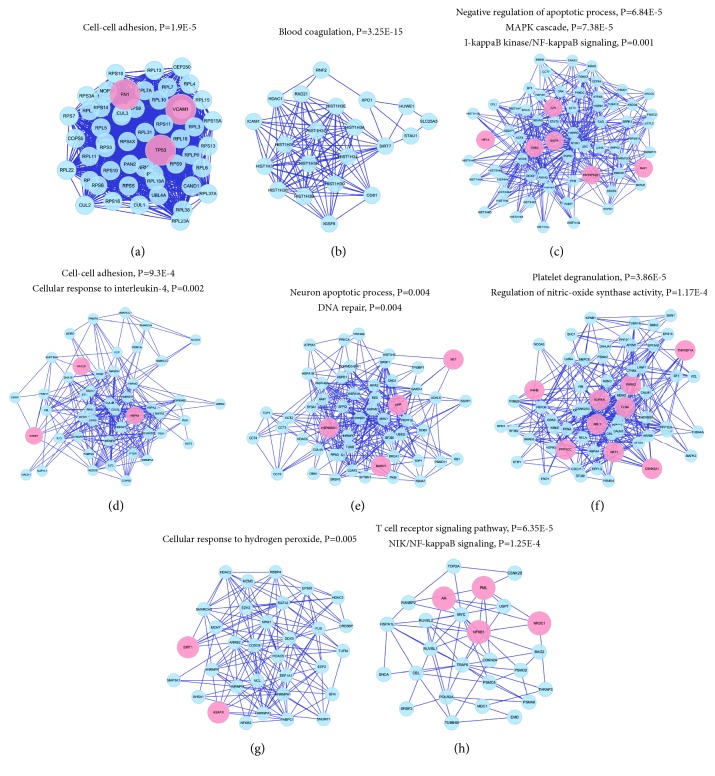
**Clusters of screened PPI networks**. a, b, c, and so on stand for clusters 1, 2, 3, and so on. Pink circles represent the seed gene related to baicalin or ischemic stroke and blue circles represent other genes in the network. Biological processes of each cluster were analyzed.

**Figure 5:**
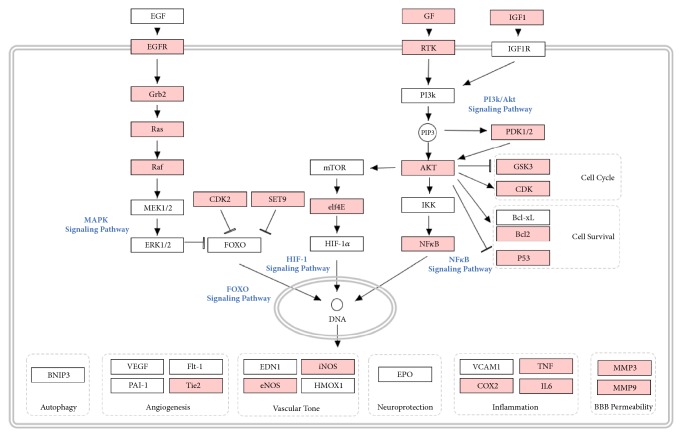
**Systematic understanding of the antistroke effects of baicalin**. In the baicalin therapeutic pathway, the pink nodes represent baicalin targets and the white nodes represent ischemic stroke targets.

Through comprehensive analysis, we obtained an integrated ischemic stroke pathway (Figure 5) based on our current knowledge of ischemic stroke pathogenesis to illuminate the integral role of baicalin in the treatment of ischemic stroke. TOP 10 KEGG signaling pathways of baicalin were obtained and constructed in bubble plot based on P-Value (Figure 3). Based on this systems-level picture, we picked and constructed five therapeutic pathways of MAPK, forkhead box (FOXO), hypoxia-inducible factor-1 (HIF-1), NF-*κ*B, and phosphoinositide 3-kinase (PI3k)/Akt signaling pathways.

## 4. Discussion

Historically, experiments in living systems were often considered as the only way to discover the potential pharmacological activities of a drug. Following advances in molecular biology, biochemistry, and pharmacology science, it has become possible to understand the relationship of drugs and molecular targets of human diseases in silico [24]. Network pharmacology helps to explore regulation of the signaling pathways with multiple channels, increase in drug efficacy and success rate of clinical trials, and decrease in the costs of drug discovery [25]. Herbal medicine and ingredients derived from plants exhibit a positive prospect in the treatment of complex diseases such as ischemic stroke due to their fewer side effects and multitarget effects [26]. Network pharmacology was successfully used in biological mechanism studies of some herbal formula [27, 28] and ingredients [29, 30]. Therefore, we used similar network pharmacology approach to understand biological mechanism of baicalin in a system level.

Firstly, absorbed, distributed, metabolized and excreted (ADME) parameters of baicalin should be considered. According to Lipinski's “rule of 5” [31] and other empirical parameters of ADME [32, 33], we introduced five ADME parameters in this study as cut-off values and found that the BBB permeability of baicalin did not meet the BBB cut-off value. Based on holistic theory, traditional Chinese medicine is thought to not only act on the central nervous system but also the cardiovascular system during ischemic stroke treatment [34]. Therefore, compounds with proper pharmacokinetic parameters but low BBB values are also accepted as promising antistroke drugs. Indeed, unlike the in silico parameters, previous pharmacokinetic data indicated that baicalin passes the BBB into the cerebral interstitial fluid [35] and accumulates in many cerebral nuclei including in the cortex, hippocampus, and striatum [36]. Therefore, some researchers consider baicalin as a potential drug for treating ischemic stroke [10], depression [37], and Alzheimer's disease [38].

Several in vivo and in vitro studies have shown that baicalin is associated with reduced brain damage and improved neurological function after ischemia-reperfusion injury. Wang et al. confirmed that baicalin can reduce cerebral infarct volume in a MCAO mouse model [39]. Other studies have considered baicalin as an antiapoptotic [40], antioxidant [11], and anti-inflammatory [41] agent. Baicalin was also found to promote the expression of neurotrophic factors [42] and regulate neurogenesis of neural progenitor cells [43], which are beneficial for neurological recovery. In the present study, we obtained 300 potential targets using an approach combining PharmMapper prediction and literature mining. We also obtained 343 targets related to ischemic stroke from four online databases. Through a topological approach, we identified a core network containing 386 nodes and 16825 edges. GO analysis suggested that baicalin plays a role in numerous stroke-related biological process, such as cell-cell adhesion, negative regulation of apoptotic process, T cell receptor signaling pathway, MAPK cascade, and others. KEGG analysis of the core PPI network indicated that the baicalin may result in multiple effects for stroke treatment by regulating multiple pathways. Based on P-Values of each enriched pathways and their relationship to ischemic stroke, five signal pathways, MAPK, FOXO, HIF-1, NF-*κ*B, and PI3k/Akt, are of most interests. Further, we constructed a signaling pathway map to further illuminate the potential molecular mechanism of baicalin in ischemic stroke from a systemic perspective.

Based on KEGG knowledge database, baicalin was thought to influence some important protein in these pathways directly or indirectly, especially three transcription factors—FOXO, HIF-1*α*, and NF-*κ*B, which were highlighted to play important roles in the anti-ischemic stroke effect of baicalin through modulating autophagy, oxidative stress, apoptosis, angiogenesis, vascular tone stability, neuroprotective effects, inflammation, and BBB permeability.

Nuclear transcription factor FOXO was thought to be involved in neuronal apoptotic signal transduction induced by hypoxic ischemia in the developing brain [44]. Indeed, FOXO is also associated with ischemia-reperfusion injury in various tissues including the heart [45] and liver [46]. Only one study investigated the relationship between baicalin and FOXO activation, implicating treatment with baicalin influenced phosphorylation/acetylation of FoxO1 via PI3K/Akt by insulin in aged rats [46]. Indeed, no literature evidence was found to discuss the relationship between baicalin and FOXO signal in ischemic rats or mice. According to KEGG knowledge, there is a complex cross-talk between PI3k/Akt, MAPK, and FOXO signal pathways. Our data showed that baicalin influenced Ras/Raf and their downstream MEK/ERK to modulate FOXO activation. Also, CDK2 was involved in the progress of FOXO modulation of baicalin [47]. Experimental studies supported relationship between baicalin and MAPK signal [48–50]. Note that activation and inhibition of baicalin on MAPK signal were all reported by researchers, which may depend on the animal model of different diseases and the dose of baicalin treatment. In the brain tissue, administration of baicalin by the dose of 200 mg/kg significantly increased the activation of ERK in gerbils after ischemia/reperfusion [51].

Evidence showed that baicalin inhibited the hypoxia-induced increase of HIF-1*α* at both the mRNA and protein levels in pulmonary artery smooth muscle cells [52]. HIF-1*α* is considered the most important transcriptional regulator of cellular responses to hypoxia. Inhibition of HIF-1*α* reduced blood-brain-barrier (BBB) damage after 2-h ischemia in a rat model of focal cerebral ischemia, and its downstream vascular endothelial growth factor (VEGF) and matrix metalloproteinase-2/9 (MMP-2/9) [53]. In addition, the role of HIF-1*α* signal pathway in ischemic brain was more complicated. Knockdown of HIF-1*α* seems impair 14 days postischemic vascular reconstruction in the ischemic rat brain [54] and protective mechanisms of HIF-1*α* are probably a consequence of promoting expressions of erythropoietin and glucose transporter 1 [55]. Therefore, the role of baicalin on HIF-1*α* signal in ischemic stroke was still controversial, and experimental studies should be designed to evaluate the role of baicalin in acute phase and recovery phase of ischemic stroke.

Furthermore, the survival of neurons after ischemia in the adult brain is associated with the activation of the PI3k/Akt signaling pathway, as inhibition of apoptosis and inflammation is a direct result of PI3k/Akt signaling [56]. Our data indicated that the neuroprotective effect of baicalin involves the PI3k/Akt pathway, which has been established by other studies [57, 58]. In a neonatal rat model of hypoxic-ischemic, baicalin was found to protect brain against hypoxic-ischemic injury by activating PI3K/Akt signaling pathway [59]. Activated Akt promotes cell survival and suppresses apoptosis partly via inhibiting glycogen synthase kinase-3 beta (GSK3*β*), an apoptosis-related molecule [60] and, activating apoptosis regulator Bcl2, an antiapoptotic molecule [61]. Interestingly, inhibited effect of baicalin on PI3k/Akt pathways was observed in leukemia cells [62] and glioblastoma cells [63]. Therefore, bidirectional regulation effect of baicalin on PI3k/Akt signaling pathway should be discussed in the future.

Inflammation is a major episode in the progression of ischemic stroke, which involves both innate and adaptive immune systems [64, 65]. Ischemic stroke-induced inflammation contributes to neurons damage and repair and has been recognized as a therapeutic target poststroke [66]. Baicalin exerts neuroprotective effects via decreasing expression of NF-*κ*B and inhibiting activation of NF-*κ*B in a dose-dependent manner [37, 67]. Baicalin was also found to downregulate the downstream signaling molecules of NF-*κ*B including iNOS, eNOS, COX2, TNF-*α*, and IL6 [68–70]. In vitro study found that baicalin inhibited the release of inflammatory factors in oxygen-glucose deprivation (OGD) brain microvascular endothelial cells (BMECs) via MAPK and NF-*κ*B signaling pathways, exhibiting a neuroprotective effect [71].

Except mentioned pathways above, literature evidence supported baicalin treating ischemic stroke via other signal pathways [72]. Recently, baicalin was found to effectively downregulate the expression of NOD2 receptor and TNF-*α* in neurons suffered ischemia-reperfusion injury [73] and inhibit activation of NLRP3 inflammasome in macrophages [74]. NLRP3 inflammasome signal pathway is considered as a novel therapeutic target for ischemic stroke [75]. Baicalin was also found to regulated mitochondrial functions in a manner dependent on AMP-activated protein kinase (AMPK) signal on ischemic condition [76]. Activation of AMPK leads to inhibition of NLRP3 inflammasome [77]. Inhibition of TLR2/4 signaling pathway was involved in its protection against ischemic injury [41, 78]. Unfortunately, these pathways were not enriched and highlighted in our study. Indeed, these pathways have a very complex cross-talk with NF-*κ*B signal, indicating NF-*κ*B may play a more important role in effect of baicalin treating ischemic stroke.

## 5. Conclusion

In the present study, we explored and discussed multiple pathways and targets of baicalin-mediated ischemic stroke treatment through a network pharmacology approach. Our data indicated that baicalin prevents ischemic stroke via multiple signaling pathways. Future studies should focus on providing experimental evidence and expanding the role of baicalin in the ischemic brain on a systems level and developing more efficient drug delivery systems to aid baicalin targeting to the brain and crossing the BBB.

## Data Availability

The data used to support the findings of this study are available from the corresponding author upon request.

## Supplementary Materials

Supplementary Materials Supplementary materials contain three tables. Supplementary Table: ADME property of baicalin. We obtained properties of absorption, distribution, metabolism, and excretion (ADME) of baicalin from TCMSP data base. Supplementary Table 2: Targets of baicalin. Targets of baicalin were predicted using PharmMapper webserver and text mining. Supplementary Table 3: Ischemic stroke related targets. We gathered ischemic stroke-related targets with the help of DrugBank, Online Mendelian Inheritance in Man (OMIM), Genetic Association Database (GAD), and Therapeutic Target Database (TTD).
