# Corrigendum to “Systematic Understanding of the Mechanism of Baicalin against Ischemic Stroke through a Network Pharmacology Approach”

**DOI:** 10.1155/2019/4520704

**Published:** 2019-04-03

**Authors:** Tian Xu, Chongyang Ma, Shuning Fan, Nan Deng, Yajun Lian, Ling Tan, Weizhe Du, Shuang Zhang, Shuling Liu, Beida Ren, Zhenhan Li, Qingguo Wang, Xueqian Wang, Fafeng Cheng

**Affiliations:** School of Traditional Chinese Medicine, Beijing University of Chinese Medicine, Beijing 100029, China

In the article titled “Systematic Understanding of the Mechanism of Baicalin against Ischemic Stroke through a Network Pharmacology Approach” [1], the names of the fourth and the twelfth authors were given incorrectly as Nang Deng and Qinguo Wang. The authors' names should have been written as Nan Deng and Qingguo Wang. The revised authors' list is shown above.
