# Supplementary material for: Systematic Understanding of the Mechanism of Baicalin against Ischemic Stroke through a Network Pharmacology Approach
Source: Evid Based Complement Alternat Med. 2018 Dec 17;2018:2582843. doi: 10.1155/2018/2582843 (PMC6311886; doi:10.1155/2018/2582843)
Supplement: Supplementary Materials — contain three tables. Supplementary Table: ADME property of baicalin. We obtained properties of absorption, distribution, metabolism, and excretion (ADME) of baicalin from TCMSP data base. Supplementary Table 2: Targets of baicalin. Targets of baicalin were predicted using PharmMapper webserver and text mining. Supplementary Table 3: Ischemic stroke related targets. We gathered ischemic stroke-related targets with the help of DrugBank, Online Mendelian Inheritance in Man (OMIM), Genetic Association Database (GAD), and Therapeutic Target Database (TTD). [file 2582843.f1.docx]

| Supplementary Table 1. ADME property of baicalin | | | | | |
| --- | --- | --- | --- | --- | --- |
| NAME | MW | AlogP | OB (%) | BBB | DL |
| Baicalin | 446.39 | 0.64 | 40.12 | -1.74 | 0.75 |

| Supplementary Table 2. Targets of baicalin | |
| --- | --- |
| GENE_NAME | GENE_SYMBOL |
| Deoxycytidine kinase | DCK |
| Uridine-cytidine kinase 2 | UCK2 |
| GTPase HRas | HRAS |
| Beta-secretase 1 | BACE1 |
| Early endosome antigen 1 | EEA1 |
| Prothrombin | F2 |
| Histo-blood group ABO system transferase | ABO |
| Glutathione S-transferase P | GSTP1 |
| Dihydrofolate reductase | DHFR |
| Ras-related protein Rap-2a | RAP2A |
| Riboflavin kinase | RFK |
| 5(3)-deoxyribonucleotidase, mitochondrial | NT5M |
| Ornithine aminotransferase, mitochondrial | OAT |
| Inosine-5-monophosphate dehydrogenase 2 | IMPDH2 |
| Matrix metalloproteinase-9 | MMP9 |
| Glucose-6-phosphate isomerase | GPI |
| 3 histone mRNA exonuclease 1 | ERI1 |
| Glutathione S-transferase theta-2 | GSTT2B |
| Ras-related protein Rab-5A | RAB5A |
| Heat shock protein HSP 90-alpha | HSP90AA1 |
| Inositol monophosphatase | IMPA1 |
| Pyruvate kinase isozymes R/L | PKLR |
| Bifunctional purine biosynthesis protein PURH | ATIC |
| Sulfotransferase family cytosolic 2B member 1 | SULT2B1 |
| Bile salt sulfotransferase | SULT2A1 |
| NAD-dependent deacetylase sirtuin-5 | SIRT5 |
| 6-phosphofructo-2-kinase/fructose-2,6-biphosphatase 1 | PFKFB1 |
| Adenosine kinase | ADK |
| Histone-lysine N-methyltransferase, H3 lysine-79 specific | DOT1L |
| ADAM 33 | ADAM33 |
| Non-secretory ribonuclease | RNASE2 |
| Pancreatic alpha-amylase | AMY2A |
| Caspase-3 | CASP3 |
| Death-associated protein kinase 1 | DAPK1 |
| RAC-alpha serine/threonine-protein kinase | AKT1 |
| ADP-ribosylation factor-like protein 5A | ARL5A |
| Sulfotransferase 1A1 | SULT1A1 |
| Maleylacetoacetate isomerase | GSTZ1 |
| Renin | REN |
| Proto-oncogene tyrosine-protein kinase LCK | LCK |
| Phosphoenolpyruvate carboxykinase, cytosolic [GTP] | PCK1 |
| Hypoxanthine-guanine phosphoribosyltransferase | HPRT1 |
| Hexokinase-1 | HK1 |
| Proto-oncogene serine/threonine-protein kinase Pim-1 | PIM1 |
| Ras-related protein Rab-11A | RAB11A |
| Bifunctional heparan sulfate N-deacetylase/N-sulfotransferase 1 | NDST1 |
| Glutathione S-transferase A1 | GSTA1 |
| Insulin receptor | INSR |
| Farnesyl pyrophosphate synthetase | FDPS |
| Phenylethanolamine N-methyltransferase | PNMT |
| Cellular retinoic acid-binding protein 2 | CRABP2 |
| Glutathione S-transferase Mu 2 | GSTM2 |
| Tryptophanyl-tRNA synthetase, cytoplasmic | WARS |
| Caspase-1 | CASP1 |
| ADP-ribosylation factor 4 | ARF4 |
| Bifunctional 3-phosphoadenosine 5-phosphosulfate synthetase 1 | PAPSS1 |
| Urokinase-type plasminogen activator | PLAU |
| Leukocyte elastase | ELANE |
| Glucokinase | GCK |
| Tyrosine-protein kinase BTK | BTK |
| Rho-related GTP-binding protein RhoE | RND3 |
| Nitric oxide synthase, inducible | NOS2 |
| Thymidylate kinase | DTYMK |
| Stromelysin-1 | MMP3 |
| Galectin-2 | LGALS2 |
| Eosinophil cationic protein | RNASE3 |
| Nucleoside diphosphate kinase B | NME2 |
| Sepiapterin reductase | SPR |
| Neprilysin | MME |
| GMP reductase 1 | GMPR |
| Mitogen-activated protein kinase 10 | MAPK10 |
| Histidine triad nucleotide-binding protein 1 | HINT1 |
| Cell division control protein 42 homolog | CDC42 |
| Triosephosphate isomerase | TPI1 |
| Interstitial collagenase | MMP1 |
| 3-phosphoinositide-dependent protein kinase 1 | PDPK1 |
| 3-hydroxy-3-methylglutaryl-coenzyme A reductase | HMGCR |
| cAMP-specific 3,5-cyclic phosphodiesterase 4B | PDE4B |
| Aldose reductase | AKR1B1 |
| Ras-related C3 botulinum toxin substrate 1 | RAC1 |
| Phenylalanine-4-hydroxylase | PAH |
| Cystathionine beta-synthase | CBS |
| Adenosylhomocysteinase | AHCY |
| Apoptotic protease-activating factor 1 | APAF1 |
| Uridine 5-monophosphate synthase | UMPS |
| Carbonic anhydrase 2 | CA2 |
| E-selectin | SELE |
| Histamine N-methyltransferase | HNMT |
| Methionine aminopeptidase 2 | METAP2 |
| Cathepsin K | CTSK |
| GMP reductase 2 | GMPR2 |
| Protein-glutamine gamma-glutamyltransferase E | TGM3 |
| Antigen peptide transporter 1 | TAP1 |
| Trifunctional purine biosynthetic protein adenosine-3 | GART |
| Glutamate carboxypeptidase 2 | FOLH1 |
| Proto-oncogene tyrosine-protein kinase Src | SRC |
| Inosine-5-monophosphate dehydrogenase 1 | IMPDH1 |
| Tyrosine-protein phosphatase non-receptor type 1 | PTPN1 |
| Cyclin-A2 | CCNA2 |
| Angiogenin | ANG |
| Kinesin-like protein KIF11 | KIF11 |
| Cell division protein kinase 2 | CDK2 |
| Serine/threonine-protein kinase 6 | AURKA |
| Adenylate kinase isoenzyme 1 | AK1 |
| Galectin-3 | LGALS3 |
| Interferon-stimulated gene 20 kDa protein | ISG20 |
| Serine/threonine-protein kinase PAK 6 | PAK6 |
| Vitamin D3 receptor | VDR |
| Estradiol 17-beta-dehydrogenase 1 | HSD17B1 |
| Triggering receptor expressed on myeloid cells 1 | TREM1 |
| Tryptophan 5-hydroxylase 1 | TPH1 |
| L-lactate dehydrogenase B chain | LDHB |
| ADP-ribosylation factor-like protein 5B | Arl5b |
| L-xylulose reductase | DCXR |
| Salivary alpha-amylase | AMY1A |
| Ras-related protein Rab-9 | RAB9A |
| Focal adhesion kinase 1 | PTK2 |
| Eukaryotic translation initiation factor 4E | EIF4E |
| Heat shock protein homolog SSE1 | SSE1 |
| Lysozyme C | LYZ |
| cAMP-specific 3,5-cyclic phosphodiesterase 4D | PDE4D |
| Mitogen-activated protein kinase 14 | MAPK14 |
| Hydroxyacyl-coenzyme A dehydrogenase, mitochondrial | HADH |
| Beta-hexosaminidase beta chain | HEXB |
| Glycogen synthase kinase-3 beta | GSK3B |
| Scavenger mRNA-decapping enzyme DcpS | DCPS |
| Fibrinogen gamma chain | FGG |
| Cathepsin B | CTSB |
| Glutathione S-transferase Mu 1 | GSTM1 |
| Glycogen phosphorylase, liver form | PYGL |
| UDP-N-acetylhexosamine pyrophosphorylase | UAP1 |
| Neutrophil collagenase | MMP8 |
| Estrogen sulfotransferase | SULT1E1 |
| Matrix metalloproteinase-16 | MMP16 |
| Signal transducer and activator of transcription 1-alpha/beta | STAT1 |
| Macrophage metalloelastase | MMP12 |
| Baculoviral IAP repeat-containing protein 7 | BIRC7 |
| Transforming growth factor beta-2 | TGFB2 |
| Glutathione S-transferase A3 | GSTA3 |
| Aldo-keto reductase family 1 member C3 | AKR1C3 |
| Coagulation factor VII | F7 |
| Glutathione reductase, mitochondrial | GSR |
| RAF proto-oncogene serine/threonine-protein kinase | RAF1 |
| Cytochrome P450 2C9 | CYP2C9 |
| Tryptase beta-2 | TPSB2 |
| Deoxyuridine 5-triphosphate nucleotidohydrolase, mitochondrial | DUT |
| Matrilysin | MMP7 |
| FK506-binding protein 3 | FKBP3 |
| Vascular endothelial growth factor receptor 2 | KDR |
| tRNA (cytosine-5-)-methyltransferase | TRDMT1 |
| ADP-ribosyl cyclase 2 | BST1 |
| Dihydroorotate dehydrogenase, mitochondrial | DHODH |
| Nicotinamide mononucleotide adenylyltransferase 3 | NMNAT3 |
| Histone acetyltransferase PCAF | KAT2B |
| Ornithine carbamoyltransferase, mitochondrial | OTC |
| Tyrosine-protein kinase HCK | HCK |
| Leukotriene A-4 hydrolase | LTA4H |
| Angiotensin-converting enzyme | ACE |
| Thyroid hormone receptor alpha | THRA |
| CD209 antigen | CD209 |
| Peptidyl-prolyl cis-trans isomerase FKBP1A | FKBP1A |
| Coagulation factor XI | F11 |
| Isovaleryl-CoA dehydrogenase, mitochondrial | IVD |
| Glutathione-requiring prostaglandin D synthase | HPGDS |
| cAMP-dependent protein kinase catalytic subunit alpha | PRKACA |
| Cell division protein kinase 7 | CDK7 |
| Betaine--homocysteine S-methyltransferase 1 | BHMT |
| Tyrosine-protein kinase JAK2 | JAK2 |
| Carboxypeptidase B | CPB1 |
| Superoxide dismutase [Mn], mitochondrial | SOD2 |
| Androgen receptor | AR |
| Glucosamine-6-phosphate isomerase | GNPDA1 |
| Galactokinase | GALK1 |
| Chitotriosidase-1 | CHIT1 |
| Interleukin-2 | IL2 |
| GTP-binding nuclear protein Ran | RAN |
| Spliceosome RNA helicase BAT1 | DDX39B |
| Dual specificity protein kinase CLK1 | CLK1 |
| Inositol-trisphosphate 3-kinase A | ITPKA |
| Cathepsin S | CTSS |
| Coagulation factor X | F10 |
| Histone deacetylase 8 | HDAC8 |
| Endoplasmic reticulum mannosyl-oligosaccharide 1,2-alpha-mannosidase | MAN1B1 |
| Arylsulfatase A | ARSA |
| Purine nucleoside phosphorylase | PNP |
| Thymidylate synthase | TYMS |
| S-methyl-5-thioadenosine phosphorylase | MTAP |
| Hepatocyte growth factor receptor | MET |
| C-C motif chemokine 5 | CCL5 |
| Serine--pyruvate aminotransferase | AGXT |
| Sorbitol dehydrogenase | SORD |
| Platelet glycoprotein Ib alpha chain | GP1BA |
| Serine/threonine-protein kinase Chk1 | CHEK1 |
| Carbonyl reductase [NADPH] 1 | CBR1 |
| NAD-dependent malic enzyme, mitochondrial | ME2 |
| UDP-glucose 4-epimerase | GALE |
| Arginase-2, mitochondrial | ARG2 |
| Complement component C8 gamma chain | C8G |
| Serine/threonine-protein phosphatase PP1-gamma catalytic subunit | PPP1CC |
| cGMP-specific 3,5-cyclic phosphodiesterase | PDE5A |
| Glutathione S-transferase omega-1 | GSTO1 |
| Phosphatidylinositol transfer protein alpha isoform | PITPNA |
| Cathepsin G | CTSG |
| Hydroxyacylglutathione hydrolase, mitochondrial | HAGH |
| Peptidyl-prolyl cis-trans isomerase FKBP1B | FKBP1B |
| Phospholipase A2, membrane associated | PLA2G2A |
| Aldo-keto reductase family 1 member C2 | AKR1C2 |
| P-selectin | SELP |
| Basic fibroblast growth factor receptor 1 | FGFR1 |
| Prostatic acid phosphatase | ACPP |
| Pyridoxine-5-phosphate oxidase | PNPO |
| Bis(5-adenosyl)-triphosphatase | FHIT |
| Adenine phosphoribosyltransferase | APRT |
| Arginase-1 | ARG1 |
| Branched-chain-amino-acid aminotransferase, mitochondrial | BCAT2 |
| Lactoylglutathione lyase | GLO1 |
| Cell division protein kinase 6 | CDK6 |
| Lithostathine-1-alpha | REG1A |
| Angiopoietin-1 receptor | TEK |
| Ephrin type-A receptor 2 | EPHA2 |
| Ephrin type-B receptor 4 | EPHB4 |
| L-serine dehydratase | SDS |
| Fatty acid-binding protein, heart | FABP3 |
| Insulin-like growth factor IA | IGF1 |
| Dipeptidase 1 | DPEP1 |
| Fructose-bisphosphate aldolase A | ALDOA |
| MAP kinase-activated protein kinase 2 | MAPKAPK2 |
| NAD(P) transhydrogenase, mitochondrial | NNT |
| Activated CDC42 kinase 1 | TNK2 |
| Glucocorticoid receptor | NR3C1 |
| Corticosteroid 11-beta-dehydrogenase isozyme 1 | HSD11B1 |
| Hepatocyte nuclear factor 4-gamma | HNF4G |
| Amine oxidase [flavin-containing] B | MAOB |
| Alcohol dehydrogenase class-3 | ADH5 |
| Spermidine synthase | SRM |
| cGMP-inhibited 3,5-cyclic phosphodiesterase B | PDE3B |
| Proto-oncogene tyrosine-protein kinase ABL1 | ABL1 |
| Heat shock cognate 71 kDa protein | HSPA8 |
| Serine hydroxymethyltransferase, cytosolic | SHMT1 |
| Growth factor receptor-bound protein 2 | GRB2 |
| Estrogen receptor | ESR1 |
| Peroxisome proliferator-activated receptor alpha | PPARA |
| Baculoviral IAP repeat-containing protein 4 | XIAP |
| Epidermal growth factor receptor | EGFR |
| Galactosylgalactosylxylosylprotein 3-beta-glucuronosyltransferase 1 | B3GAT1 |
| Alpha-tocopherol transfer protein | TTPA |
| Complement factor B | CFB |
| Nuclear receptor subfamily 1 group I member 2 | NR1I2 |
| Nicotinamide mononucleotide adenylyltransferase 1 | NMNAT1 |
| Thyroid hormone receptor beta | THRB |
| Bone morphogenetic protein 7 | BMP7 |
| Thymidine kinase, cytosolic | TK1 |
| Cytidine deaminase | CDA |
| Transthyretin | TTR |
| ADAM 17 | ADAM17 |
| GTP-binding protein Rheb | RHEB |
| Complement factor D | CFD |
| Sex hormone-binding globulin | SHBG |
| Protein-L-isoaspartate(D-aspartate) O-methyltransferase | PCMT1 |
| Ferrochelatase, mitochondrial | FECH |
| Kynurenine--oxoglutarate transaminase 1 | KYAT1 |
| C-1-tetrahydrofolate synthase, cytoplasmic | MTHFD1 |
| Pleckstrin homology domain-containing family A member 4 | PLEKHA4 |
| Tyrosine-protein kinase ITK/TSK | ITK |
| Nuclear receptor ROR-alpha | RORA |
| Fatty acid-binding protein, epidermal | FABP5 |
| Retinoic acid receptor gamma | RARG |
| Dipeptidyl peptidase 4 | DPP4 |
| S-adenosylmethionine decarboxylase proenzyme | AMD1 |
| Lanosterol synthase | LSS |
| Serum albumin | ALB |
| 72 kDa type IV collagenase | MMP2 |
| Cathepsin F | CTSF |
| Retinoic acid receptor RXR-beta | RXRB |
| Neutrophil gelatinase-associated lipocalin | LCN2 |
| Phosphopantothenoylcysteine decarboxylase | PPCDC |
| Glucose-6-phosphate 1-dehydrogenase | G6PD |
| Retinoic acid receptor alpha | RARA |
| Mitogen-activated protein kinase 12 | MAPK12 |
| BAG family molecular chaperone regulator 1 | BAG1 |
| Casein kinase II subunit alpha | CSNK2A1 |
| Liver carboxylesterase 1 | CES1 |
| Histone-lysine N-methyltransferase SETD7 | SETD7 |
| Retinoic acid receptor RXR-alpha | RXRA |
| Retinoic acid receptor beta | RARB |
| Bile acid receptor | NR1H4 |
| Macrophage migration inhibitory factor | MIF |
| Mast/stem cell growth factor receptor | KIT |
| Seprase | FAP |
| Ribonuclease 4 | RNASE4 |
| Glucosylceramidase | GBA |
| Serum amyloid P-component | APCS |
| Progesterone receptor | PGR |
| Nitric oxide synthase, endothelial | NOS3 |
| Pyruvate dehydrogenase E1 component subunit beta, mitochondrial | PDHB |
| Casein kinase I isoform gamma-2 | CSNK1G2 |
| Cytochrome P450 1A2 | CYP1A2 |
| Cytochrome P450 1A1 | CYP1A1 |
| Tumor necrosis factor | TNF |
| Heme oxygenase 1 | HMOX1 |
| G1/S-specific cyclin-D1 | CCND1 |
| Interleukin-6 | IL6 |
| Acetylcholinesterase | ACHE |
| Aryl hydrocarbon receptor | AHR |
| Apoptosis regulator BAX | BAX |
| Pro-epidermal growth factor | EGF |
| Transcription factor HES-1 | HES1 |
| Hairy/enhancer-of-split related with YRPW motif protein 1 | HEY1 |
| Hairy/enhancer-of-split related with YRPW motif protein 2 | HEY2 |
| Neurogenic locus notch homolog protein 1 | NOTCH1 |
| Prostaglandin G/H synthase 2 | PTGS2 |
| Toll-like receptor 4 | TLR4 |
| Thioredoxin-interacting protein | TXNIP |
| ABC transporter C family member 2 | ABCC2 |
| Arachidonate 5-lipoxygenase | ALOX5 |
| Cyclic AMP-dependent transcription factor ATF-2 | ATF2 |
| Apoptosis regulator Bcl-2 | BCL2 |
| Collagen alpha-2(I) chain | COL1A2 |
| ETS domain-containing protein Elk-1 | ELK1 |
| Serine/threonine-protein kinase/endoribonuclease IRE1 | ERN1 |
| Aspartate aminotransferase | GOT1 |
| Alanine aminotransferase 1 | GPT |
| Interleukin-1 beta | IL1B |
| Myeloid differentiation primary response protein MyD88 | MYD88 |
| NF-kappa-B inhibitor alpha | NFKBIA |
| NACHT, LRR and PYD domains-containing protein 3 | NLRP3 |
| Pyruvate kinase | PKM |
| Peroxisome proliferator-activated receptor gamma | PPARG |
| Proline-rich membrane anchor 1 | PRIMA1 |
| Transcription factor p65 | RELA |
| Transforming growth factor beta-1 | TGFB1 |
| Cellular tumor antigen p53 | TP53 |

| Supplementary Table 3. Ischemic stroke related targets | | |
| --- | --- | --- |
| GENE_SYMBOL | GENE_NAME | Source |
| PTGS2 | Prostaglandin G/H synthase 2 | TTD |
| GABBR2 | Gamma-aminobutyric acid receptor | TTD |
| HTR3A | 5-hydroxytryptamine receptor | TTD |
| GRIA2 | Glutamate receptor AMPA subtype | TTD |
| NMDAR | NMDA receptor | TTD |
| KCNMB1 | Calcium-activated potassium channel | TTD |
| PDE1A | Calcium/calmodulin-dependent 3',5'-cyclic nucleotide phosphodiesterase 1 | TTD |
| PARP1 | Poly [ADP-ribose] polymerase-1 | TTD |
| F2 | Thrombin | TTD |
| GLRA1 | Glycine receptor | TTD |
| F2R | Proteinase activated receptor 1 | TTD |
| MMP2 | 72 kDa type IV collagenase | TTD |
| ITGB2 | Integrin beta-2 | TTD |
| FLNA | Filamin A | TTD |
| IRF1 | Interferon regulatory factor 1 | TTD |
| PDE4D | cAMP-specific 3',5'-cyclic phosphodiesterase 4D | OMIM |
| GP1BA | Platelet glycoprotein Ib alpha chain | OMIM |
| EPHX2 | Bifunctional epoxide hydrolase 2 | OMIM |
| MTHFR | Methylenetetrahydrofolate reductase | OMIM |
| APOE | Apolipoprotein E | OMIM |
| PROZ | Vitamin K-dependent protein Z | OMIM |
| VCAM1 | Vascular cell adhesion protein 1 | OMIM |
| PDGFRA | Platelet-derived growth factor receptor alpha | OMIM |
| NPPB | Natriuretic peptides B | OMIM |
| PDGFC | Platelet-derived growth factor C | OMIM |
| NOTCH3 | Neurogenic locus notch homolog protein 3 | OMIM |
| SELP | P-selectin | OMIM |
| F2 | Prothrombin | OMIM |
| ABCC8 | ATP-binding cassette sub-family C member 8 ABC transporter C family member 8 | OMIM |
| RNF213 | E3 ubiquitin-protein ligase RNF213 | OMIM |
| TET2 | Tetraspanin-2 Methylcytosine dioxygenase TET2 | OMIM |
| F5 | Coagulation factor V | OMIM |
| TNFRSF1A | Tumor necrosis factor receptor superfamily member 1A | OMIM |
| ACTA2 | Actin, aortic smooth muscle | OMIM |
| COL4A1 | Collagen alpha-1(IV) chain | OMIM |
| THBD | Thrombomodulin | OMIM |
| KCNK2 | Potassium channel subfamily K member 2 | OMIM |
| CECR1 | Adenosine deaminase 2 | OMIM |
| LDLR | Low-density lipoprotein receptor | OMIM |
| IL4 | Interleukin-4 | OMIM |
| SREBF1 | Sterol regulatory element-binding protein 1 | OMIM |
| INSIG1 | Insulin-induced gene protein Insulin-induced gene 1 protein | OMIM |
| BAK1 | BRASSINOSTEROID INSENSITIVE 1-associated receptor kinase 1 Bcl-2 homologous antagonist/killer | OMIM |
| PLG | Plasminogen | OMIM |
| BARD1 | BRCA1-associated RING domain protein 1 | OMIM |
| GUCY1A3 | Guanylate cyclase soluble subunit alpha-1 | OMIM |
| PCSK9 | Proprotein convertase subtilisin/kexin type 9 | OMIM |
| DDIT4 | DNA damage-inducible transcript 4 protein | OMIM |
| ALOX5AP | Arachidonate 5-lipoxygenase-activating protein | OMIM |
| SIRT2 | NAD-dependent protein deacetylase NAD-dependent protein deacetylase sirtuin-2 | OMIM |
| AQP4 | Aquaporin-4 | OMIM |
| CBS | Cystathionine beta-synthase Cystathionine beta-synthase-like protein | OMIM |
| CFH | Complement factor H | OMIM |
| MYBPC3 | Myosin-binding protein C, cardiac-type | OMIM |
| COL3A1 | Collagen alpha-1(III) chain | OMIM |
| ARL6IP6 | ADP-ribosylation factor-like protein 6-interacting protein 6 | OMIM |
| PROS1 | Vitamin K-dependent protein S | OMIM |
| ATP7B | Copper-transporting ATPase 2 | OMIM |
| SELENOS | Selenoprotein S | OMIM |
| NOS1 | Nitric oxide synthase, brain Nitric oxide synthase | OMIM |
| MMP9 | Matrix metalloproteinase-9 | OMIM |
| GRIN2B | Glutamate receptor ionotropic, NMDA 2B | OMIM |
| APOLD1 | Apolipoprotein L domain-containing protein 1 | OMIM |
| SCG2 | Secretogranin-2 | OMIM |
| PARK2 | E3 ubiquitin-protein ligase parkin | OMIM |
| DLG4 | Disks large homolog 4 | OMIM |
| HTRA1 | Serine protease HTRA1 | OMIM |
| HYOU1 | Hypoxia up-regulated protein 1 | OMIM |
| EPO | Erythropoietin | OMIM |
| UCP2 | Mitochondrial uncoupling protein 2 | OMIM |
| ACCN2 | Acid-sensing ion channel 1 | OMIM |
| TNF | Tumor necrosis factor | OMIM |
| TNFRSF1B | Tumor necrosis factor receptor superfamily member 1B | OMIM |
| SLC1A2 | Amino acid transporter Excitatory amino acid transporter 2 | OMIM |
| LGMN | Legumain | OMIM |
| SET | Protein SET | OMIM |
| AGAP2 | Arf-GAP with GTPase, ANK repeat and PH domain-containing protein 2 | OMIM |
| GRIN1 | Glutamate receptor ionotropic, NMDA 1 | OMIM |
| VEGFA | Vascular endothelial growth factor A | OMIM |
| HSF1 | Heat stress transcription factor A-1a Heat shock factor protein 1 Heat shock factor protein | OMIM |
| FGB | Fibrinogen beta chain | OMIM |
| DAPK1 | Death-associated protein kinase 1 | OMIM |
| APP | Amyloid-beta A4 protein | OMIM |
| YRDC | YrdC domain-containing protein, mitochondrial | OMIM |
| GH1 | Somatotropin Auxin-induced protein | OMIM |
| GRIN2A | Glutamate receptor ionotropic, NMDA 2A | OMIM |
| AKT1S1 | Proline-rich AKT1 substrate 1 | OMIM |
| PRKAA1 | Non-specific serine/threonine protein kinase 5'-AMP-activated protein kinase catalytic subunit alpha-1 | OMIM |
| MIF | Macrophage migration inhibitory factor | OMIM |
| PRKCE | Protein kinase C epsilon type Protein kinase C | OMIM |
| GRIN3A | Glutamate receptor ionotropic, NMDA 3A | OMIM |
| KCNE2 | Potassium voltage-gated channel subfamily E member 2 | OMIM |
| ADORA2A | Adenosine receptor A2 Adenosine receptor A2a | OMIM |
| NF1 | Neurofibromin | OMIM |
| TRPA1 | Transient receptor potential cation channel subfamily A member 1 | OMIM |
| HIF1A | Hypoxia-inducible factor 1-alpha | OMIM |
| CRP | Pentaxin C-reactive protein | OMIM |
| BCL2L11 | Bcl-2-like protein 11 | OMIM |
| TRIM2 | Tripartite motif-containing protein 2 | OMIM |
| CD4 | T-cell surface glycoprotein CD4 | OMIM |
| AGTR1 | Type-1 angiotensin II receptor | OMIM |
| MAPKAPK2 | MAP kinase-activated protein kinase 2 | OMIM |
| CXCL12 | Stromal cell-derived factor 1 | OMIM |
| KDR | Vascular endothelial growth factor receptor 2 Transcription factor PRE6 | OMIM |
| AKT1 | Non-specific serine/threonine protein kinase RAC-alpha serine/threonine-protein kinase | OMIM |
| MAPK10 | Mitogen-activated protein kinase Mitogen-activated protein kinase 10 | OMIM |
| STC1 | Stanniocalcin-1 | OMIM |
| CDK5 | Cyclin-dependent-like kinase 5 | OMIM |
| PPARGC1A | Peroxisome proliferator-activated receptor gamma coactivator 1-alpha | OMIM |
| PGF | Placenta growth factor | OMIM |
| EGLN1 | Egl nine homolog 1 | OMIM |
| CTSL | Cathepsin L1 | OMIM |
| NDNF | Protein NDNF | OMIM |
| SHMT2 | Serine hydroxymethyltransferase Serine hydroxymethyltransferase, mitochondrial | OMIM |
| GLDC | Glycine cleavage system P protein Glycine dehydrogenase (decarboxylating), mitochondrial | OMIM |
| S100B | Protein S100 Protein S100-B | OMIM |
| THBS1 | Thrombospondin-1 | OMIM |
| AQP1 | Aquaporin-1 | OMIM |
| P2RX5 | p2X purinoceptor P2X purinoceptor P2X purinoceptor 5 | OMIM |
| ACCN3 | Acid-sensing ion channel 3 | OMIM |
| C3 | Replication enhancer Complement C3 Complement C3 alpha chain Replication enhancer protein | OMIM |
| C3AR1 | C3a anaphylatoxin chemotactic receptor | OMIM |
| NGB | Neuroglobin | OMIM |
| KCNMB1 | Calcium-activated potassium channel subunit beta-1 | OMIM |
| GPR17 | Uracil nucleotide/cysteinyl leukotriene receptor | OMIM |
| TLR4 | Toll-like receptor 4 | OMIM |
| ESR1 | Estrogen receptor Ethylene-responsive transcription factor ESR1 | OMIM |
| SERPINE1 | Plasminogen activator inhibitor 1 | OMIM |
| CLU | Clusterin | OMIM |
| TCF17 | TRANSCRIPTION FACTOR 17 | OMIM |
| HAVCR1 | Hepatitis A virus cellular receptor 1 | OMIM |
| IL20 | Interleukin-20 | OMIM |
| APIP | Methylthioribulose-1-phosphate dehydratase | OMIM |
| HMOX1 | Heme oxygenase Heme oxygenase 1 | OMIM |
| FNDC1 | Fibronectin type III domain-containing protein 1 | OMIM |
| JAK2 | Tyrosine-protein kinase Tyrosine-protein kinase JAK2 | OMIM |
| NFKB1 | Nuclear factor NF-kappa-B p105 subunit | OMIM |
| PLP2 | Phosducin-like protein 2 Proteolipid protein 2 | OMIM |
| MMP2 | 72 kDa type IV collagenase | OMIM |
| G6PD | Glucose-6-phosphate 1-dehydrogenase | OMIM |
| HNRNPA2B1 | Heterogeneous nuclear ribonucleoproteins A2/B1 | OMIM |
| RGCC | Regulator of cell cycle RGCC | OMIM |
| HNRNPL | Heterogeneous nuclear ribonucleoprotein L | OMIM |
| SLC2A1 | Solute carrier family 2, facilitated glucose transporter member 1 | OMIM |
| NPY | Pro-neuropeptide Y | OMIM |
| SLC1A3 | Amino acid transporter Excitatory amino acid transporter 1 | OMIM |
| BDKRB1 | B1 bradykinin receptor | OMIM |
| ADORA3 | Adenosine receptor A3 Transmembrane domain-containing protein TMIGD3 | OMIM |
| BDKRB2 | B2 bradykinin receptor | OMIM |
| CAMK2A | Calcium/calmodulin-dependent protein kinase type II subunit alpha | OMIM |
| CLIC4 | Chloride intracellular channel protein 4 Chloride intracellular channel protein | OMIM |
| CRYAA | Alpha-crystallin A chain | OMIM |
| VEGFB | Vascular endothelial growth factor B | OMIM |
| CCL21 | C-C motif chemokine 21 | OMIM |
| ADIPOQ | Adiponectin | OMIM |
| BCL2 | Apoptosis regulator Bcl-2 | OMIM |
| PDGFB | Platelet-derived growth factor subunit B | OMIM |
| FGF2 | Fibroblast growth factor Fibroblast growth factor 2 | OMIM |
| LCN2 | Neutrophil gelatinase-associated lipocalin | OMIM |
| NDRG4 | Protein NDRG4 | OMIM |
| PARP1 | Poly [ADP-ribose] polymerase Poly [ADP-ribose] polymerase 1 | OMIM |
| SOD1 | Superoxide dismutase [Cu-Zn] Superoxide dismutase | OMIM |
| BNIP3 | BCL2/adenovirus E1B 19 kDa protein-interacting protein 3 | OMIM |
| BACE1 | Beta-secretase 1 | OMIM |
| GGA3 | ADP-ribosylation factor-binding protein GGA3 | OMIM |
| PPIF | Peptidyl-prolyl cis-trans isomerase Peptidyl-prolyl cis-trans isomerase F, mitochondrial | OMIM |
| SLC8A3 | Sodium/calcium exchanger 3 | OMIM |
| EDIL3 | EGF-like repeat and discoidin I-like domain-containing protein 3 | OMIM |
| SAV1 | Protein salvador homolog 1 | OMIM |
| SUCNR1 | Succinate receptor 1 | OMIM |
| ZC3H12A | Endoribonuclease ZC3H12A Ribonuclease ZC3H12A | OMIM |
| PYGM | Alpha-1,4 glucan phosphorylase Glycogen phosphorylase, muscle form | OMIM |
| C1QR1 | Complement component C1q receptor | OMIM |
| FN1 | Fibronectin | OMIM |
| TMBIM6 | Bax inhibitor 1 | OMIM |
| CTNNA3 | Catenin alpha-3 | OMIM |
| EIF2S1 | Eukaryotic translation initiation factor 2 subunit 1 | OMIM |
| HTRA2 | Serine protease HTRA2, mitochondrial | OMIM |
| CD300LB | CMRF35-like molecule 7 | OMIM |
| SHC3 | SHC-transforming protein 3 | OMIM |
| CASP2 | CASP-like protein Casparian strip membrane protein 2 Caspase-2 | OMIM |
| SOD2 | Superoxide dismutase [Mn], mitochondrial Superoxide dismutase | OMIM |
| OGFOD1 | Prolyl 3-hydroxylase OGFOD1 | OMIM |
| IGHD2 | Somatotropin | OMIM |
| PIK3R1 | Phosphatidylinositol 3-kinase regulatory subunit alpha | OMIM |
| SERPINF1 | Pigment epithelium-derived factor | OMIM |
| PLAT | Plasminogen activator Tissue-type plasminogen activator | OMIM |
| P4HB | Protein disulfide-isomerase | OMIM |
| HNF1B | Hepatocyte nuclear factor 1-beta | OMIM |
| DES | Desmin 9-divinyl ether synthase | OMIM |
| HSD11B1 | Corticosteroid 11-beta-dehydrogenase isozyme 1 | OMIM |
| GRK5 | G protein-coupled receptor kinase G protein-coupled receptor kinase 5 | OMIM |
| GRM3 | Metabotropic glutamate receptor 3 | OMIM |
| NTN1 | Netrin-1 | OMIM |
| AR | Androgen receptor | OMIM |
| LAMA4 | Laminin subunit alpha-4 | OMIM |
| CAMP | Cathelicidin antimicrobial peptide Cathelicidin-7 Antimicrobial protein CAP18 Cathelicidin-2 | OMIM |
| LRP1 | Low-density lipoprotein receptor-related protein 1 Protein LATERAL ROOT PRIMORDIUM 1 Prolow-density lipoprotein receptor-related protein 1 Exosome complex protein LRP1 | OMIM |
| TLR2 | Toll-like receptor Toll-like receptor 2 | OMIM |
| PARG | Poly(ADP-ribose) glycohydrolase | OMIM |
| KMO | Kynurenine 3-monooxygenase | OMIM |
| ADAMTS13 | A disintegrin and metalloproteinase with thrombospondin motifs 13 | OMIM |
| STIM1 | Stromal interaction molecule 1 | OMIM |
| OLIG2 | Oligodendrocyte transcription factor 2 | OMIM |
| EGLN2 | Egl nine homolog 2 | OMIM |
| TXNRD2 | Thioredoxin reductase 2, mitochondrial | OMIM |
| SLC8B1 | Mitochondrial sodium/calcium exchanger protein | OMIM |
| ZNF667 | Zinc finger protein 667 | OMIM |
| LMF1 | Lipase maturation factor Lipase maturation factor 1 | OMIM |
| SIRT1 | NAD-dependent protein deacetylase sirtuin-1 | OMIM |
| PMAIP1 | Phorbol-12-myristate-13-acetate-induced protein 1 | OMIM |
| CDK5RAP3 | CDK5 regulatory subunit-associated protein 3 | OMIM |
| H2AFX | Histone H2A Histone H2AX | OMIM |
| MBL2 | Mannose-binding protein C Mannose-binding protein | OMIM |
| MC1R | Melanocyte-stimulating hormone receptor | OMIM |
| LIF | Leukemia inhibitory factor | OMIM |
| CXCR4 | C-X-C chemokine receptor type 4 | OMIM |
| FLT1 | Vascular endothelial growth factor receptor 1 | OMIM |
| SPP1 | Osteopontin Probable sucrose-phosphatase 1 COMPASS component SPP1 Lipid phosphate phosphatase delta | OMIM |
| REN | Replication enhancer Renin | OMIM |
| SOD3 | Extracellular superoxide dismutase [Cu-Zn] | OMIM |
| TTN | Titin | OMIM |
| COL15A1 | Collagen alpha-1(XV) chain | OMIM |
| CASP3 | Caspase-3 Casparian strip membrane protein 3 CASP-like protein | OMIM |
| THBS4 | Thrombospondin-4 | OMIM |
| IL18 | Interleukin-18 | OMIM |
| MTOR | Serine/threonine-protein kinase mTOR Serine/threonine-protein kinase TOR | OMIM |
| GRINA | Protein lifeguard 1 | OMIM |
| GRIN2C | Glutamate receptor ionotropic, NMDA 2C | OMIM |
| NRG1 | Transcriptional regulator NRG1 Pro-neuregulin-1, membrane-bound isoform | OMIM |
| PML | Protein PML Retinoic acid receptor alpha | OMIM |
| PSEN1 | Presenilin Presenilin-1 | OMIM |
| SERPINA3 | Alpha-1-antichymotrypsin Serpin A3-5 | OMIM |
| ATP7A | Copper-transporting ATPase 1 | OMIM |
| TMSB4X | Thymosin beta-4 | OMIM |
| CD40LG | CD40 ligand | OMIM |
| WT1 | Wilms tumor protein homolog Wilms tumor protein | OMIM |
| NPC1 | NPC intracellular cholesterol transporter 1 Non-specific phospholipase C1 | OMIM |
| GRIN2D | Glutamate receptor ionotropic, NMDA 2D | OMIM |
| TMEM123 | Porimin | OMIM |
| ANKRD1 | Ankyrin repeat domain-containing protein 1 | OMIM |
| PLA2G3 | Group 3 secretory phospholipase A2 | OMIM |
| ADPGK | ADP-dependent glucokinase | OMIM |
| KL | Klotho | OMIM |
| S1PR2 | Sphingosine 1-phosphate receptor 2 | OMIM |
| DLL4 | Delta-like protein Delta-like protein 4 | OMIM |
| PAPPA | Pappalysin-1 | OMIM |
| HBB | Hemoglobin subunit beta | OMIM |
| CA4 | Carbonic anhydrase 4 | OMIM |
| CTNNA1 | Catenin alpha-1 | OMIM |
| OLR1 | Oxidized low-density lipoprotein receptor 1 | OMIM |
| PC | Pyruvate carboxylase Pyruvate carboxylase, mitochondrial | OMIM |
| CD36 | Platelet glycoprotein 4 | OMIM |
| PHKA1 | Phosphorylase b kinase regulatory subunit Phosphorylase b kinase regulatory subunit alpha, skeletal muscle isoform | OMIM |
| ANG | Angiogenin | OMIM |
| APOC3 | Apolipoprotein C-III | OMIM |
| MVK | Mevalonate kinase | OMIM |
| APOC2 | Apolipoprotein C-II | OMIM |
| TAX1BP3 | Tax1-binding protein 3 | OMIM |
| TNFRSF1A | Tumor necrosis factor receptor superfamily member 1A | OMIM |
| PDE4D | Phosphodiesterase cAMP-specific 3',5'-cyclic phosphodiesterase 4D | OMIM |
| NOS3 | Nitric oxide synthase Nitric oxide synthase, endothelial | OMIM |
| ACTA2 | Actin, aortic smooth muscle | OMIM |
| SIRT2 | NAD-dependent protein deacetylase sirtuin-2 | OMIM |
| FGB | Fibrinogen beta chain | GAD |
| F7 | Coagulation factor VII | GAD |
| F5 | Coagulation factor V | GAD |
| F2 | Prothrombin | GAD |
| CYBA | Cytochrome b-245 light chain | GAD |
| CPB2 | Carboxypeptidase B2 | GAD |
| CBS | Cystathionine beta-synthase | GAD |
| APOA1 | Apolipoprotein A-I | GAD |
| AGTR1 | Type-1 angiotensin II receptor | GAD |
| ADH1B | Alcohol dehydrogenase 1B | GAD |
| ACE | Angiotensin-converting enzyme | GAD |
| TNF | Tumor necrosis factor | GAD |
| THBD | Thrombomodulin | GAD |
| TH | Tyrosine 3-monooxygenase | GAD |
| TAF1 | Transcription initiation factor TFIID subunit 1 | GAD |
| SERPINE1 | Plasminogen activator inhibitor 1 | GAD |
| PTGIS | Prostacyclin synthase | GAD |
| PROZ | Vitamin K-dependent protein Z | GAD |
| PON1 | Serum paraoxonase/arylesterase 1 | GAD |
| NR3C1 | Glucocorticoid receptor | GAD |
| NOTCH3 | Neurogenic locus notch homolog protein 3 | GAD |
| LPL | Lipoprotein lipase | GAD |
| LDLR | Low-density lipoprotein receptor | GAD |
| IL1A | Interleukin-1 alpha | GAD |
| TP53 | Cellular tumor antigen p53 | GAD |
| PAFAH1B1 | Platelet-activating factor acetylhydrolase IB subunit alpha | GAD |
| MPO | Myeloperoxidase | GAD |
| APOE | Apolipoprotein E | GAD |
| CCL11 | Eotaxin | GAD |
| CCL5 | C-C motif chemokine 5 | GAD |
| COMT | Catechol O-methyltransferase | GAD |
| EDNRA | Endothelin-1 receptor | GAD |
| F13A1 | Coagulation factor XIII A chain | GAD |
| IL6 | Interleukin-6 | GAD |
| ITGB2 | Integrin beta-2 | GAD |
| JAK2 | Tyrosine-protein kinase JAK2 | GAD |
| LCAT | Phosphatidylcholine-sterol acyltransferase | GAD |
| LIPG | Endothelial lipase | GAD |
| MTHFR | Methylenetetrahydrofolate reductase | GAD |
| RETN | Resistin | GAD |
| TBXA2R | Thromboxane A2 receptor | GAD |
| TNFSF4 | Tumor necrosis factor ligand superfamily member 4 | GAD |
| EDN1 | Endothelin-1 | GAD |
| PRKCH | Protein kinase C eta type | GAD |
| P2RY2 | P2Y purinoceptor 2 | GAD |
| NCF1 | Neutrophil cytosol factor 1 | GAD |
| MMP3 | Stromelysin-1 | GAD |
| LIMK1 | LIM domain kinase 1 | GAD |
| KCNN4 | Intermediate conductance calcium-activated potassium channel protein 4 | GAD |
| APOH | Beta-2-glycoprotein 1 | GAD |
| IRAK1 | Interleukin-1 receptor-associated kinase 1 | GAD |
| APOB | Apolipoprotein B receptor | GAD |
| IL4 | Interleukin-4 receptor subunit alpha | GAD |
| GP1BA | Platelet glycoprotein Ib alpha chain | GAD |
| GJA5 | Gap junction alpha-5 protein | GAD |
| ABCA1 | ATP-binding cassette sub-family A member 1 | GAD |
| CYP4F2 | Phylloquinone omega-hydroxylase CYP4F2 | GAD |
| CYP4A11 | Cytochrome P450 4A11 | GAD |
| PRKCG | Protein kinase C gamma type | TTD |
| DAPK1 | Death-associated protein kinase 1 | TTD |
| CA10 | Carbonic anhydrase-related protein 10 | TTD |
| CALCA | Calcitonin gene-related peptide 1 | TTD |
| LAMB1 | Laminin subunit beta-1 | TTD |
| PKC | Protein kinase C | TTD |
| CALCB | Calcitonin gene-related peptide 2 | TTD |
| STK36 | Serine/threonine-protein kinase 36 | TTD |
| SLC29A4 | Equilibrative nucleoside transporter 4 | TTD |
| HTR1B | 5-hydroxytryptamine receptor 1B | TTD |
| CIB1 | Calcium and integrin-binding protein 1 | TTD |
| EPHB2 | Ephrin type-B receptor 2 | TTD |
| APP | Amyloid beta A4 protein | TTD |
| JUN | activator protein 1 | TTD |
| HTR1B | 5-hydroxytryptamine receptor 1 | TTD |
| ASIC3 | Acid-sensing ion channel 3 | TTD |
| GRIN2B | Glutamate receptor ionotropic, NMDA 2B | TTD |
| ASIC1 | Acid-sensing ion channel 1 | TTD |
| HIF1A | Hypoxia-inducible factor 1-alpha | TTD |
| GRIN1 | Glutamate (NMDA) receptor | TTD |
| KCNB1 | Potassium voltage-gated channel subfamily B member 1 | TTD |
